# Sero – epidemiology of brucellosis in people and their livestock: A linked human – animal cross-sectional study in a pastoralist community in Kenya

**DOI:** 10.3389/fvets.2022.1031639

**Published:** 2022-11-18

**Authors:** Josphat Muema, Harriet Oboge, Nyamai Mutono, Anita Makori, Julius Oyugi, Zipporah Bukania, Joseph Njuguna, Christine Jost, Brian Ogoti, Sylvia Omulo, S. M. Thumbi

**Affiliations:** ^1^Institute of Tropical and Infectious Diseases, University of Nairobi, Nairobi, Kenya; ^2^Washington State University Global Health Program - Kenya, Nairobi, Kenya; ^3^Feed the Future Innovation Lab for Animal Health, Washington State University, Pullman, WA, United States; ^4^Centre for Epidemiological Modeling and Analysis, University of Nairobi, Nairobi, Kenya; ^5^Center for Public Health Research, Kenya Medical Research Institute, Nairobi, Kenya; ^6^Food and Agriculture Organization of the United Nations, Nairobi, Kenya; ^7^United States Agency for International Development's Bureau for Humanitarian Assistance (USAID/BHA), Washington, DC, United States; ^8^Global Health Support Initiative III, Social Solutions International, Washington, DC, United States; ^9^Paul G. Allen School for Global Health, Washington State University, Pullman, WA, United States; ^10^South African Center for Epidemiological Modeling Analysis, Stellenbosch, South Africa; ^11^Institute of Immunology and Infection Research, University of Edinburgh, Edinburgh, United Kingdom

**Keywords:** brucellosis, sero-epidemiology, pastoralists, livestock, Kenya

## Abstract

**Background:**

Brucellosis is associated with massive livestock production losses and human morbidity worldwide. Efforts to control brucellosis among pastoralist communities are limited by scarce data on the prevalence and risk factors for exposure despite the high human-animal interactions in these communities. This study simultaneously assessed the seroprevalence of brucellosis and associated factors of exposure among pastoralists and their livestock in same households.

**Methods:**

We conducted a cross-sectional study in pastoralist communities in Marsabit County – Kenya. A total of 1,074 women and 225 children participated and provided blood samples. Blood was also drawn from 1,876 goats, 322 sheep and 189 camels. Blood samples were collected to be screened for the presence of anti-Brucella IgG antibodies using indirect IgG Enzyme-Linked Immunosorbent Assay (ELISA) kits. Further, Individual, household and herd-level epidemiological information were captured using a structured questionnaire. Group differences were compared using the Pearson's Chi-square test, and *p*-values < 0.05 considered statistically significant. Generalized mixed-effects multivariable logistic human and animal models using administrative ward as the random effect was used to determine variables correlated to the outcome.

**Results:**

Household-level seropositivity was 12.7% (95% CI: 10.7–14.8). The individual human seroprevalence was 10.8% (9.1–12.6) with higher seroprevalence among women than children (12.4 vs. 3.1%, *p* < 0.001). Herd-level seroprevalence was 26.1% (23.7–28.7) and 19.2% (17.6–20.8) among individual animals. Goats had the highest seroprevalence 23.1% (21.2 – 25.1), followed by sheep 6.8% (4.3–10.2) and camels 1.1% (0.1–3.8). Goats and sheep had a higher risk of exposure OR = 3.8 (95% CI 2.4–6.7, *p* < 0.001) and 2.8 (1.2–5.6, *p* < 0.007), respectively relative to camels. Human and animal seroprevalence were significantly associated (OR = 1.8, [95%CI: 1.23–2.58], *p* = 0.002). Herd seroprevalence varied by household head education (OR = 2.45, [1.67–3.61, *p* < 0.001]) and herd size (1.01, [1.00–1.01], *p* < 0.001).

**Conclusions:**

The current study showed evidence that brucellosis is endemic in this pastoralist setting and there is a significant association between animal and human brucellosis seropositivity at household level representing a potential occupational risk. Public health sensitization and sustained human and animal brucellosis screening are required.

## Background

Brucellosis is an endemic neglected zoonotic disease and a major cause of morbidity in humans and livestock in low- and middle-income countries (1). Brucellosis is associated with significant economic burden (2) and is estimated to account for income losses of 6–10% per animal (3). Humans contract brucellosis primarily by consuming unpasteurized dairy products or undercooked meat, or by handling of aborted fetuses or placenta of infected animals (4). Animals exposure occurs primarily by animal contact with infected aborted material, ingestion of contaminated pastures or milk. Sexual transmission can occur through natural mating or artificial insemination. In humans, brucellosis manifests as a debilitating illness and undulating fever (5) while in domestic ruminants it is mainly characterized by reproductive disorders including abortions, infertility and retained placenta.

While brucellosis is among the top ten priority zoonotic diseases in Kenya, efforts toward its control and prevention are lagging (6). Previous studies have reported varied estimates of brucellosis prevalence in the country ranging from 0 to 47% (7–9) among humans and 1 to 38% among animals, with communities inhabited by nomadic pastoralists recording the highest estimates (7, 10, 11). Few have reported brucellosis infections in sympatric human and animal populations in Kenya. Nomadic pastoralists are most vulnerable to brucellosis due to high interactions with their livestock and the consumption of their products (12). Nevertheless, studies among these communities are scarce and the factors associated with exposure can vary widely. We simultaneously estimated the seroprevalence of brucellosis among people and livestock living in the same households and identified putative risk factors for exposure.

## Methods

### Study area

**Figure 1:**
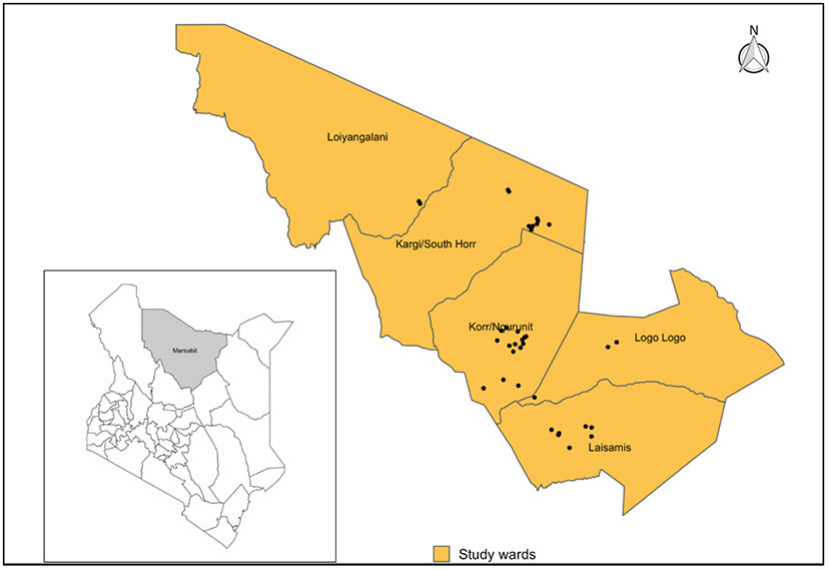
Map showing the position of Marsabit County within Kenya **(Left)**, Laisamis sub-county and wards included in the study indicating all sampled villages (Black dots). Shapefile source: GADM.

This study was conducted in Laisamis sub-county of Marsabit county in northern Kenya, within Logologo, Laisamis, Kargi, Korr and Loiyangalani wards where nomadic pastoralism is practiced (Figure 1). Livestock herds are primarily composed of cattle, sheep, goats and camels (13). However, the study was conducted at a time when the region was experiencing drought and cattle had migrated out of the study area in search of water and pasture, hence we did not sample cattle. These communities are dependent on their animals for subsistence and they live in close contact to their animals including women who herd small ruminants.

### Study population

The study piggy-backed on a larger research project, livestock for health (L4H) project, which is a cluster randomized controlled trial investigating how providing supplementary feeds to livestock during dry periods (when pastoralists migrate) impacts maternal and child nutrition in northern kenya. The study population for the L4H project was composed of women of reproductive age, children <5 years and livestock providing milk to the households. This population was chosen because women of child bearing age, especially pregnant and lactating women and children <5 years of age are the most nutritionally vulnerable group and are a good indicator of a household nutritional status. We investigated the burden of brucellosis in these same population since high prevalences of brucellosis have been reported in similar pastoral production systems in kenya (7) and also due to the severe, debilitating and chronic nature of brucellosis (14), we wanted to determine if its associated with the high rates of malnutrition reported in women and children in this setting. Consequently, our sampling population did not include the whole population and comprised women of reproductive age, children <5 years and lactating livestock (camels, sheep and goats) providing milk to this households. Cattle were not sampled because they had migrated outside the study area in search of water and pasture.

### Study design, sample size, and sampling strategy

This cross-sectional study was conducted between September and November 2019. Multi-stage cluster sampling was conducted to select potential enrolees. A list of all sublocations within the five wards was generated and 12 sublocations were randomly selected. A list of all villages within each sublocations was then generated and used as a sampling frame to randomly select three villages per sublocation. In each village, households with a lactating animal, a child l < 5 years and woman of reproductive age were identified for possible inclusion in the study.

Households were the primary sampling units while individuals (children, women or lactating animals) were secondary sampling units. A household herd was defined as aggregate flocks (cattle, goats, sheep, and camels) managed under the same household. We assumed that household herds share common risk factors for disease and that disease distribution within the herd was homogenous. Sample size calculation was based on the formula for sample size determination when herds, flocks or other aggregates of animals are the sampling units and taking into account herd effects to achieve high herd level sensitivity and specificity while also accounting for test imperfections as the ELISA kits used had <100% sensitivity and specificity (15, 16).


$$\begin{array}{l} n={\left(1.96/d\right)}^{2}\;X\\ \frac{\left[(Se_{agg}\;x\;P_{\text{exp}})+(1-Sp_{agg})(1-P_{\text{exp}})][(1-Se_{agg}\;x\;P_{\text{exp}})-(1-Sp_{agg})(l\;-P_{\text{exp}})\right]}{{\left(Se_{agg}+Sp_{agg}-1\right)}^{2}} \end{array}$$

We applied an expected herd prevalence (*P*_*ex*_*)* of 50%, a desired absolute precision (*d)* of 5%, and aggregate test sensitivity (Se_agg_) and specificity (Sp_agg_) of 95 and 99%, respectively, to obtain a minimum sample size of 960 households. We chose the 50% prevalence because it provides the largest sample size for given values of absolute error.

In each household herd, up to three lactating animals per species were chosen by systematic random selection. A sampling interval number was obtained by dividing the total number of lactating animals per species by number of animals to be sampled within the herd. The first animal was then randomly selected followed by every nth animal until the sample size was attained. In each household herd, all lactating animals per species were grouped together and numbered using animal marker pens and random numbers assigned by dividing the total number of lactating animals per species by three (3) to create the interval of selection. Animals bearing the random number were selected for blood sample collection. For human participants, children and women within households that consented to participate in the L4H study were enrolled for blood collection.

### Data collection

Household and herd-level data were abstracted from the L4H baseline survey data. These data included household demographic characteristics, herd health, herd management practices, livestock production system, location (ward) and human nutrition status. Individual-level factors (animal and human) were collected using a structured questionnaire, which was administered to an adult household respondent (≥18 years). These factors included species, age, sex, physiological status, and history of reproductive disorders for animals, and participant type (mother or child), age, sex, and physiological status for humans.

### Sample collection

Human and animal blood specimens were collected via venipuncture by trained nurses and animal health technicians, respectively. Human samples were collected in plain 5 mL vacutainers while animal samples were collected in 10 mL vacutainers. For the human samples, 2.5 mL of blood was collected from children and 4 mL from women while for the animal samples, 8 mL of blood was collected from goats, sheep and camels. Samples tubes were barcoded and allowed to stand for 15 min to allow for clot separation. Clotted samples were then transported to a field laboratory in cooler boxes within 6 h of collection.

### Laboratory processing

At the lab, samples were accessioned, then centrifuged at 3,000 *x*g for 10 min. Harvested sera were transferred into 2 mL cryovials labeled with the corresponding barcode IDs. Sera were stored at −20°C until transported to the University of Nairobi Institute of Tropical and Infectious Diseases (UNITID) laboratory where they were stored at −80°C until tested.

Prior to testing, samples were thawed at room temperature. Indirect ELISA kits—PrioCHECK™ Brucella Ab 2.0 Strip Kit, (Themo Fisher Scientific, UK) and IBL-America Brucella IgG ELISA (Immuno-Biological Laboratories Inc, USA) were used to screen for *Brucella* spp IgG antibodies in animal and human sera, respectively. Testing proceeded according to manufacturer's instructions. Animal sample ODs were read at 450 nm and interpreted as positive or negative based on percent positivity (PP) cut-off values of <25 or >25, respectively. Human sample ODs were read at 405 nm and a reference wavelength of 630 nm. Results were interpreted based on cut-off values calculated using test control results as described in the manufacturer's quality control certificate recommendations.

### Data management and statistical analysis

Field data were electronically captured using the CommCare^®^ mobile application and downloaded as CSV files. Laboratory data were captured using an excel template. Data were cleaned, merged and analyzed using R version 3.6.2 (17). Socio-demographic characteristics of the study population were summarized as frequencies or proportions. Individual and herd-level seroprevalence were calculated to estimate brucella species exposure levels within the study area. A herd was considered positive if at least one animal in the herd was positive.

Group differences were compared using the Pearson's Chi-square test, and *p*-values < 0.05 considered statistically significant. Multivariable logistic regression analyses were performed for human and animal models to identify the factors associated with brucellosis seropositivity. The location ward was included as a random effect to account for clustering at the ward level. Odds ratio (OR) values with 95% confidence intervals (CIs) confirmed associations (or lack thereof) between brucellosis seropositivity and potential risk factors.

The independent predictor variables were selected based on their biological plausibility and/or documented association with brucellosis seropositivity. The significance level was set at *P* ≤ 0.2, and independent variable(s) that met this criterion were included in the multivariable mixed-effects logistic regression model. In the human models these included age, sex, physiological status, occupation, education level, location (ward) and nutritional status. For animal models, they included species, location (ward), reproductive disorders, household head occupation, household head education level and grazing distance. In this context, a household head was defined as an adult person, male or female, who is responsible for the organization and care of the household, and has overall decision making authority in the household. Predictor variables were added to the respective models and a stepwise variable selection approach using the Akaike Information Criterion (AIC) algorithm used to determine the best fitting model. The model with the lowest AIC value was selected. Model diagnostics including calculating scaled residuals, mapping residuals, and testing for dispersion and spatial autocorrelation of residuals were conducted prior to selecting the final models. Model building assumed family binomial with logit link functions.

### Ethical statement

This study was approved by the Kenya Medical Research Institute Scientific and Ethics Review Unit (KEMRI/SERU/CGHR/02-09/3755) and the Kenyatta National Hospital/University of Nairobi Ethics and Research Committee (KNH-ERC/A/69-P850/10/2019). Written informed consent was obtained from adult participants and children's guardians prior to their enrollment into the study. All animal owners provided written informed consent before specimen collection. Animal restraint and sampling were conducted in a manner to minimize discomfort to animals and enhance personal safety, and were conducted by trained animal technicians and veterinary surgeons following the World Organization for Animal Health (WOAH) guidelines for use of animals in research and education (18).

## Results

### Socio-demographic characteristics of human and animal study population

Of 1,734 households enrolled in the larger L4H study, 1,050 (61%) consented to participate in this brucellosis study. From these 1,050 households, a total of 1,299 participants were enrolled and provided samples,1,074 (83%) of whom were women and 255 (17%) children. The average age of enrolled women was 29 years (range: 17–46), while that of children was 23 months (range: 5–42). Among women, 905 (84.3%) were lactating, most (988, 92%) had no formal education and 728 (68%) practiced livestock herding as their primary occupation. Among the children recruited, 145 (64%) were female and 80 (36%) were male. All households owned at least one livestock type with 96% owning goats, 92% sheep, 68% camels and 43% cattle. The average number of animals owned per household was seven goats, six sheep, three camels, and three cattle. Together, 1,244 household herds were included and 2,387 blood samples collected from 1,876 (78%) goats, 322 (14%) sheep and 189 (8%) camels. No cattle were sampled as the few cattle kept by the communities were in dry season grazing areas.

### Brucellosis seroprevalence in women and children

Of the 1,050 enrolled households, 133 had ≥1 seropositive participant, resulting in a household-level seroprevalence of 12.7% (95% CI: 10.7–14.8). Individual human-level seroprevalence was 10.8% (9.1–12.6), with a higher seroprevalence observed in women than in children (12.4 vs. 3.1%, *p* < 0.001). No significant difference in seroprevalence between male and female children (4 vs. 3%, *p* < 0.682). Seroprevalence varied with socio-demographic characteristics (Table 1).

**Table 1:** Brucellosis seroprevalence in women and children by sociodemographic characteristics and results of univariable analysis.

**Variable**	**Women (*****N*** = **1,074)**			**Children (*****N*** = **225)**		
	**% (n/N)**	**95% CI**	***p*-value**	**% (n/N)**	**95% CI**	***p*-value**
**Occupation**						
Herding	12.9 (94/728)	10.6–15.6	0.658	-	-	
Employed	11.3 (39/346)	8.1–15.1		-	-	
**Physiological status**						
Lactating	10.5 (104/905)	4.9–18.9	0.088	-	-	
Non-lactating	17.2 (29/169)	11.8–23.7		-	-	
Education level^*^						
Formal education	10.5 (9/86)	4.9–18.9	0.573	0 (0/17)	-	0.442
No formal education	12.5 (124/988)	10.5–14.6		3.4 (7/208)	1.4–6.8	
**Location (ward)**						
Kargi	7.6 (16/209)	4.4–12.1	0.169	0 (0/8)	-	0.606
Korr	12.4 (53/426)	9.5–15.9		1.5 (1/66)	0.0–8.2	
Laisamis	14.2 (37/260)	10.2–19.1		2.8 (2/72)	0.3–9.7	
Logologo	15.3 (25/163)	10.2–21.8		5.1 (4/79)	1.4–12.5	
Loiyangalani	12.5 (2/16)	1.6**–**38.4		-	-	
**Nutritional status**						
Malnourished	8.6 (11/128)	4.4–14.9	0.165	2.2 (1/45)	0.1–11.8	0.701
Healthy	12.9 (122/946)	10.8–15.2		3.3 (6/180)	1.2–7.1	

* For children, this refers to mother's education level.

### Brucellosis seroprevalence in animals

Out of 1,244 herds sampled, 325 had at least one seropositive animal resulting in a herd seroprevalence of 26.1% (95% CI: 23.7–28.7). The overall animal-level brucellosis seroprevalence was 19.2% (17.6 – 20.8), with seroprevalence varying by animal type; 23.1% (21.2–25.1) in goats, 6.8% (4.3–10.2) in sheep and 1.1% (0.1–3.8) in camels. Seroprevalence in animals varied by sociodemographic characteristics (Table 2).

**Table 2:** Brucellosis seroprevalence in animals by socio-demographic characteristics.

**Variable**	**Seroprevalence %(n/N)**	**95% CI**	***p*-value**
**Location (Ward)**			
Kargi	10 (41/395)	7.6–13.8	<0.001
Korr	12 (113/931)	10.1–14.4	
Laisamis	24 (122/520)	19.8–27.4	
Logologo	41 (142/350)	35.4–45.9	
Loiyangalani	20 (39/191)	14.9–26.8	
**Livestock type**			
Goats	23 (433/1,876)	21.2–25.1	<0.001
Sheep	7 (22/322)	4.3–10.2	
Camels	1 (2/189)	0.1–3.8	
**Reproductive disorders**			
No	18 (301/1,641)	16.5–20.3	0.139
Yes	21 (156/746)	18.1–24.0	
**Household head occupation**			
Herding	19 (337/1,789)	17.1–20.7	0.508
Employed	20 (120/598)	16.9–23.5	
**Household head education**			
No formal education	19 (360/1,882)	17.4–20.9	0.002
Formal education	29 (49/167)	22.6–36.9	
**Grazing distance**			
<5 km	29 (127/66)	16.3–22.4	0.059
5–10 km	22 (162/746)	18.8–24.9	
>10 km	17 (168/979)	14.9–19.7	

### Factors associated with brucellosis seropositivity in women and children

At Household level, we observed significant associations at the household level between brucellosis exposure in people and their livestock (OR = 1.7, 95%CI: 1.2–2.5, *p* = 0.002). None of the potential risk factors (age, sex, occupation, physiological status, geographical location and nutrition status) included in the models were significantly associated with seropositivity among women or children (*p* > 0.05).

### Factors associated with brucellosis seroprevalence in animals

At herd level, goat herds (OR = 3.86, 95%CI: 2.34 – 6.73, *p* < 0.001) and sheep flocks (OR = 3.02, 1.42–5.91, *p* = 0.003) had higher odds of being brucellosis seropositive compared to camel herds. There was a significant association between seropositive herds and seropositive households (OR = 1.8, 1.23–2.58, *p* = 0.002). Herds owned by household heads with formal education had higher odds of being brucellosis seropositive (OR = 2.45, 1.67–3.61, *p* < 0.001) compared to those owned by household heads with no formal education. There were significantly higher odds of brucellosis among animal herds from larger herds sizes compared to smaller ones (OR = 1.006, 95%CI 1.003 – 1.009, *p* < 0.001; Table 3).

**Table 3:** Herd-level factors associated with brucellosis seropositivity.

**Variable**	**OR (CI)**	***p-*value**
**Livestock type**		
Goats	3.856 (2.344–6.728)	<0.001
Sheep	3.017 (1.416–5.914)	0.003
Camels	Ref	
**Household seropositivity**		
Positive	1.785 (1.228–2.576)	0.002
Negative	Ref	
**Household head education**		
Formal education	2.454 (1.670–3.606)	<0.001
No formal education	Ref	
Herd size	1.006 (1.003–1.009)	<0.001

At the individual animal level, goats (OR = 3.8, 95%CI: 2.4–6.7, p <0.001) and sheep (OR = 2.8, 1.2–5.7, p = 0.007) had significantly higher odds of being brucellosis seropositive compared to camels (Table 4).

**Table 4:** Animal-level factors associated with brucellosis seropositivity.

**Variable**	**OR (95% CI)**	***p*-value**
**Livestock type**		
Camels	Ref	
Goats	3.88 (2.37–6.75)	<0.001
Sheep	2.76 (1.17–5.65)	0.007
**Household head education**		
No formal education	Ref	
Formal education	1.38 (0.94–2.00)	0.091

## Discussion

This “One Health” sero-epidemiology study of brucellosis among people and their livestock from a predominantly pastoral community in Kenya indicated high prevalence of brucellosis in people and domestic ruminants from the same households. By simultaneously studying both people and their livestock, we examine the associations between exposure status in animals and people and find a significant association between animal and human brucellosis seropositivity at household level. Further, we explored factors associated with increased risk of brucella species exposure in both human and domestic ruminants' population and highlighted the implications of our findings to disease burden, spread and control strategies.

A systematic review of brucellosis in Kenya estimates that the national human brucellosis seroprevalence is 3%, compared to 10.3% among pastoralist communities (11), supporting the estimate of 11% in our study community. Nevertheless, our estimate is lower than those reported in other pastoralist communities, which range between 14 and 36% (7, 8, 19). The high seroprevalence observed in pastoralist communities are attributed to increased frequency of human contact with infected livestock and consumption of unpasteurized dairy products (7, 20, 21). Infected animals shed bacteria in milk and parturition materials, which increases the probability of human infection during human-animal interactions (22). This may also explain why women in our study—the majority of whom were herders—had higher seropositivity than children. Further, assuming that brucellosis is endemic in this setting, older persons in general are likely to have more exposures over time compared to children.

The higher seroprevalence in animals compared to humans in our study (19 vs. 11%) suggests a higher likelihood of exposure among animals than humans. In nomadic production systems, large herds interact in communal grazing lands and watering points, increasing the likelihood of disease transmission (23). Nevertheless, these results contrast those of an earlier study in Kenya which reported seroprevalence levels of 3.5% in animals and 35.8% in humans (8). The observed differences may be attributable to differences in our study populations. We sampled children <5 years and reproductive-age women in a community that practices a pure pastoral production system, while the earlier study sampled the general population in a community that practices irrigated agricultural production.

The seroprevalence of brucellosis among animals varied by species. There were 4 and 3-fold higher odds of brucellosis seropositivity in goats and sheep, respectively, compared to camels. Similar results have been reported in two pastoral settings in Kenya which found a higher likelihood of exposure among small ruminants compared to other species (7). This could be because small ruminants mainly graze near homesteads were abortions are more likely to occur, increasing their risk of exposure. Alternatively, these differences may be due to varying susceptibility to *Brucella* spp. among different animal species. Further research is required to determine the drivers of species differences in *Brucella spp*. infection in this setting.

Seropositivity at the herd level increased with increasing herd size. Similar relationships have been reported in previous studies (8, 24–26) and could be explained by the higher probability of mixing between infected and susceptible animals in large herds (27). Additionally, the pastoral production system increases the probability of animal contact between and within herds due to communal grazing system, and concentration of animals at common watering points (27).

We found higher odds of brucellosis seropositivity among livestock from households with formally educated than non-formally educated household heads, contrary to findings by Njenga et al. (28). Formally educated household heads are more likely to own larger herds due to their higher economic status which may have contributed to the observed higher brucellosis prevalence.

Our study found a significant association between human and animal seropositivity at the household level, with the odds of human sero-positivity being 1.8 times higher in households with a seropositive animal compared to those without. These results indicate that seropositivity in humans depends on human–animal contact (23, 29) and that animals are reservoirs and sources of brucellosis for humans (7, 8). Unlike in our study, studies conducted in Togo and Mongolia found no associations between human and animal brucellosis seropositivity (30, 31). This may be attributed to the village-level sampling employed in the two studies. Further, the study in Mongolia did not require human and animal sampling from the same households.

We found no correlation between brucellosis seropositivity in humans and malnutrition. Nevertheless, since we tested for exposure to *Brucella* spp., we could not distinguish past exposure and active brucellosis infections. Therefore, we cannot rule out the influence of brucellosis infection on human nutritional status either directly or indirectly.

This study had few limitations. Our study population comprised of lactating animals, children < 5 years and women of reproductive age. While these populations provided data on exposure levels for this population, they may not be representative of the general population. The lack of sampling cattle, which is also a key species kept in this setting limited the generalizability of our results. The cross-sectional nature of our study limited our assessment of temporal variations in brucellosis seropositivity. We used an indirect IgG ELISA to test the presence of antibodies against *Brucella* spp. and could not distinguish between past exposure and active infections. Further, failure to also consider to use IgM ELISA kit in addition to the IgG may have led to failure to detect some positive cases that had acute phase of the disease and hence our reported seroprevalence may not be the true prevalence of the disease due to potential misclassification bias.

A key strength of our study is the use of One Health concept by simultaneously assessing brucellosis exposure in people and their livestock. In this case, we find evidence of household level association between levels of exposure to brucellosis in livestock and people.

## Conclusions

Our study provides evidence that brucellosis is endemic in pastoralist settings and there is a significant association between animal and human brucellosis seropositivity at household level. These data can contribute to formulating targeted control interventions that focus on the risk factors that are unique to such communities. Public health sensitization and sustained human and animal brucellosis screening are required. To better assess the true burden of brucellosis, its transmission dynamics and socio-economic impact, further studies are warranted. Coupling linked human-animal study approaches with the use of molecular diagnostic techniques to speciate circulating *Brucella* spp. may provide detailed information to guide brucellosis control and prevention interventions.

## Data availability statement

The raw data supporting the conclusions of this article will be made available by the authors, without undue reservation.

## Ethics statement

The studies involving human participants were reviewed and approved by Kenya Medical Research Institute Scientific and Ethics Review Unit and Kenyatta National Hospital/University of Nairobi Ethics and Research Committee. Written informed consent to participate in this study was provided by the participants' legal guardian/next of kin. Written informed consent was obtained from the owners for the participation of their animals in this study.

## Author contributions

Designed and planned the study protocol, drafted the manuscript, and participated in data analysis: JM, JO, ZB, NM, AM, BO, HO, JN, CJ, and ST. Contributed to data and sample collection and ELISA work: JM, BO, HO, NM, AM, and ST. All authors revised and approved the final manuscript.

## Funding

The research was made possible through the support provided by the Office of Technical and Program Quality, Bureau for Humanitarian Assistance, U.S. Agency for International Development (720FDA18IO00035). Research reported in this publication was supported by the Fogarty International Center and the Institute of Allergy and Infectious Diseases of the National Institutes of Health under Award Number D43TW011519 as part of JM doctoral fellowship.

## Conflict of interest

The authors declare that the research was conducted in the absence of any commercial or financial relationships that could be construed as a potential conflict of interest.

## Publisher's note

All claims expressed in this article are solely those of the authors and do not necessarily represent those of their affiliated organizations, or those of the publisher, the editors and the reviewers. Any product that may be evaluated in this article, or claim that may be made by its manufacturer, is not guaranteed or endorsed by the publisher.

## Author disclaimer

The opinions expressed in this paper are those of the author(s) and do not necessarily reflect the views of the U.S. Agency for International Development or the US Government. The content is solely the responsibility of the authors and does not necessarily represent the official views of the National Institutes of Health.
